# Diagnosis of Mucopolysaccharidoses

**DOI:** 10.3390/diagnostics10030172

**Published:** 2020-03-22

**Authors:** Francyne Kubaski, Fabiano de Oliveira Poswar, Kristiane Michelin-Tirelli, Maira Graeff Burin, Diana Rojas-Málaga, Ana Carolina Brusius-Facchin, Sandra Leistner-Segal, Roberto Giugliani

**Affiliations:** 1 Postgraduate Program in Genetics and Molecular Biology, UFRGS, Porto Alegre 91501-970, Brazil; fkubaski@udel.edu (F.K.); fposwar@hcpa.edu.br (F.d.O.P.); diana.rojas.malaga@gmail.com (D.R.-M.); 2 Medical Genetics Service, HCPA, Porto Alegre 90035-903, Brazil; ktirelli@hcpa.edu.br (K.M.-T.); mburin@hcpa.edu.br (M.G.B.); afacchin@hcpa.edu.br (A.C.B.-F.); ssegal@hcpa.edu.br (S.L.-S.); 3 INAGEMP, Porto Alegre 90035-903, Brazil; 4 Biodiscovery Research Group, Experimental Research Center, HCPA, Porto Alegre 90035-903, Brazil; 5 Postgraduate Program in Medicine, Clinical Sciences, UFRGS, Porto Alegre 90035-003, Brazil

**Keywords:** mucopolysaccharidoses, glycosaminoglycans, enzyme replacement therapy, tandem mass spectrometry, newborn screening.

## Abstract

The mucopolysaccharidoses (MPSs) include 11 different conditions caused by specific enzyme deficiencies in the degradation pathway of glycosaminoglycans (GAGs). Although most MPS types present increased levels of GAGs in tissues, including blood and urine, diagnosis is challenging as specific enzyme assays are needed for the correct diagnosis. Enzyme assays are usually performed in blood, with some samples (as leukocytes) providing a final diagnosis, while others (such as dried blood spots) still being considered as screening methods. The identification of variants in the specific genes that encode each MPS-related enzyme is helpful for diagnosis confirmation (when needed), carrier detection, genetic counseling, prenatal diagnosis (preferably in combination with enzyme assays) and phenotype prediction. Although the usual diagnostic flow in high-risk patients starts with the measurement of urinary GAGs, it continues with specific enzyme assays and is completed with mutation identification; there is a growing trend to have genotype-based investigations performed at the beginning of the investigation. In such cases, confirmation of pathogenicity of the variants identified should be confirmed by measurement of enzyme activity and/or identification and/or quantification of GAG species. As there is a growing number of countries performing newborn screening for MPS diseases, the investigation of a low enzyme activity by the measurement of GAG species concentration and identification of gene mutations in the same DBS sample is recommended before the suspicion of MPS is taken to the family. With specific therapies already available for most MPS patients, and with clinical trials in progress for many conditions, the specific diagnosis of MPS as early as possible is becoming increasingly necessary. In this review, we describe traditional and the most up to date diagnostic methods for mucopolysaccharidoses.

## 1. Introduction

The mucopolysaccharidoses (MPSs) comprises 11 lysosomal diseases in which there is a deficiency in a specific step of the degradation of glycosaminoglycans (GAGs). This deficiency leads to storage of GAGs in tissues and to a range of clinical consequences, which may include CNS impairment, depending on the specific MPS type [1,2]. Each MPS is clinically heterogeneous, with severe and attenuated cases within each MPS type, a fact that may be related to small variations in the residual enzyme activity, conditioned by the genetic variation present in the patient [3]. 

The diagnosis of MPSs is currently based on the evaluation of GAGs, measurement of enzyme activities and identification of genetic variants. The correct diagnosis enables specific therapeutic measures (available for most MPS patients) to be taken, and also phenotype prediction, carrier identification, genetic counseling, and prenatal diagnosis. As therapy outcomes seem to be better when the disease is identified early in life, screening for MPS in high-risk groups and even newborn screening programs for selected MPS types are taking place [2,4,5,6,7,8].

This chapter provides an overview of the tools available for the diagnosis of MPS, and it discusses the use of these tools in the investigation of suspected patients, in the screening of high-risk groups and in newborn screening. 

## 2. Clinical Suspicion and High-Risk Groups

Where no newborn screening program is available, physicians face the challenge of recognizing an MPS patient in the early stages of the disease, before irreversible damage has developed. In many cases, patients with MPSs and other LDs (lysosomal disorders) may present specific signs that are highly suggestive of the diagnosis [9]. Nevertheless, delayed diagnoses are still common and even efforts to increase awareness may have a limited impact [10,11]. In this section, we will discuss the strategies for the selective screening of MPS in high-risk groups.

In some case reports, a presenting manifestation was the key to the suspicion of MPS [12,13,14]. In those cases, MPS-related signs and symptoms were already present (see Table 1), although the diagnosis was not considered before. On the other hand, it seems very unlikely that patients presenting with an isolated manifestation will be diagnosed with MPS if not under a selective screening program and/or a simultaneous screening for several disorders with overlapping signs and symptoms. Among the possible isolated manifestations of MPS, developmental delay and osteoarticular manifestations may be relevant for screening programs that aim at the early diagnosis of these conditions.

In the early stages of neuronopathic forms of MPS, particularly MPS III, developmental delay or regression of skills may be the only prominent manifestation. In a study that included 944 unrelated patients with an intellectual disability of unknown cause, seven patients were diagnosed with an inborn error of metabolism, none of them with MPS [15]. Regardless of the low diagnostic yield, urinary GAGs are usually included in many protocols for the investigation of intellectual disability in the first-line investigation [16].

Patients with attenuated phenotypes of MPS are likely to consult rheumatologists and orthopedists before being diagnosed, due to several osteoarticular manifestations, including stiff joints and carpal tunnel syndrome [17,18]. A study that screened 55 patients with osteoarticular problems of unknown etiology found one with attenuated MPS II [19]. In another study, 188 patients with juvenile idiopathic arthritis were screened for MPS IX, all with normal results [20]. Finally, a recent study in 425 adult subjects with carpal tunnel syndrome performed in Denmark failed to find any cases of MPS I, MPS II or MPS VI [21].

The low diagnostic yields of those studies that focused on single manifestations may be explained by the rarity of MPS as compared to other causes of intellectual disability and osteoarticular problems. Considering the rarity of MPS and the nonspecific nature of isolated manifestations, investigating patients with a combination of manifestations is also a reasonable approach. For instance, in a study from Malaysia, high-risk patients were selected based on having at least two of a list of eight signs and symptoms related to MPS [22]. Among the 60 patients investigated, 15 of them were diagnosed with an MPS disorder. As there is a significant variation in the estimated birth prevalence of the mucopolysaccharidoses across the world [23,24], it is also important to emphasize that studies describing the diagnostic yield of the screening of high-risk groups may not be reproducible in different populations. 

To aid clinicians in recognizing high-risk groups that should be investigated by selective screening, the combination of signs and symptoms of MPS may be summarized in suspicion scores or mnemonics. Using the data of patients included in the Hunter Outcome Survey (HOS), HUNTER, was developed, a mnemonic screening tool for MPS II (Hernia/Hearing, Unusual faces, Nasal obstruction, Tongue and Tonsils, Enlarged liver and spleen, Respiration/Range of Motion). The mnemonic score was weighted according to the likelihood of the sign or symptom to be specifically associated with Hunter syndrome. A score of six or more was found to have a 95% accuracy for the diagnosis of MPS II, as assessed in the HOS sample, although that was not validated in the clinical setting [25].

Focusing on the cardiologists, a list for the identification of systemic and cardiovascular “red flags” for the clinical suspicion of MPS in children was proposed: valve diseases, cardiomyopathy; hump/spinal column malformations; and the triad inguinal hernia + respiratory infections + hip dysplasia [26]. For adults, the same authors proposed as red flags: non-inflammatory aortopathy; corneal opacity; and retinitis. Other similar red flags were proposed for pediatric endocrinologists when evaluating children with short stature [12].

Besides the signs and symptoms, information on family history is also very important for the suspicion of MPS. Since MPS types are either autosomal recessive or X-linked, a male-to-male transmission of the phenotype makes it unlikely to be caused by an MPS disorder. Table 2 summarizes high-risk groups, for whom an investigation for mucopolysaccharidoses and other differential diagnoses is suggested. 

## 3. Biomarkers

Biomarkers are analytes that can be measured and used to indicate a pathological or physiological process, thus they allow discrimination within disease vs. non-pathological events. If well-chosen, a biomarker can be helpful for diagnosis, prognosis, and they might also be useful to monitor therapeutic efficacy [27,28,29].

At present, with the advent of robust tools such as next-generation sequencing and tandem mass spectrometry, our knowledge of disease mechanisms and pathophysiology has increased, allowing the identification of biomarkers that have a higher probability of being informative [30].

As MPSs are primarily associated to the GAG storage, GAGs are a natural biomarker for these diseases [1,31]. There are different subclasses of GAGs that can be accumulated according to the specific enzymatic defect: dermatan sulfate (DS), heparan sulfate (HS), keratan sulfate (KS) and hyaluronan (HA) [1,32]. This accumulation can also vary according to the residual levels of enzyme activity, type of genetic variant, and environmental factors [33,34].

**Figure 1:**
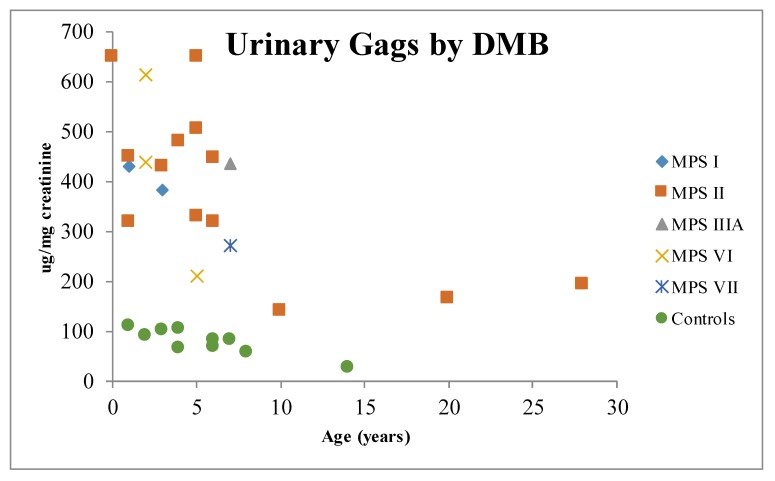
Distribution of urinary glycosaminoglycans by age. MPS I: mucopolysaccharidosis type I; MPS II: mucopolysaccharidosis type II; MPS IIIA: mucopolysaccharidosis type IIIA; MPS VI: mucopolysaccharidosis type VI; MPS VII: mucopolysaccharidosis type VII; DMB: dimethyl methylene blue.

**Figure 2:**
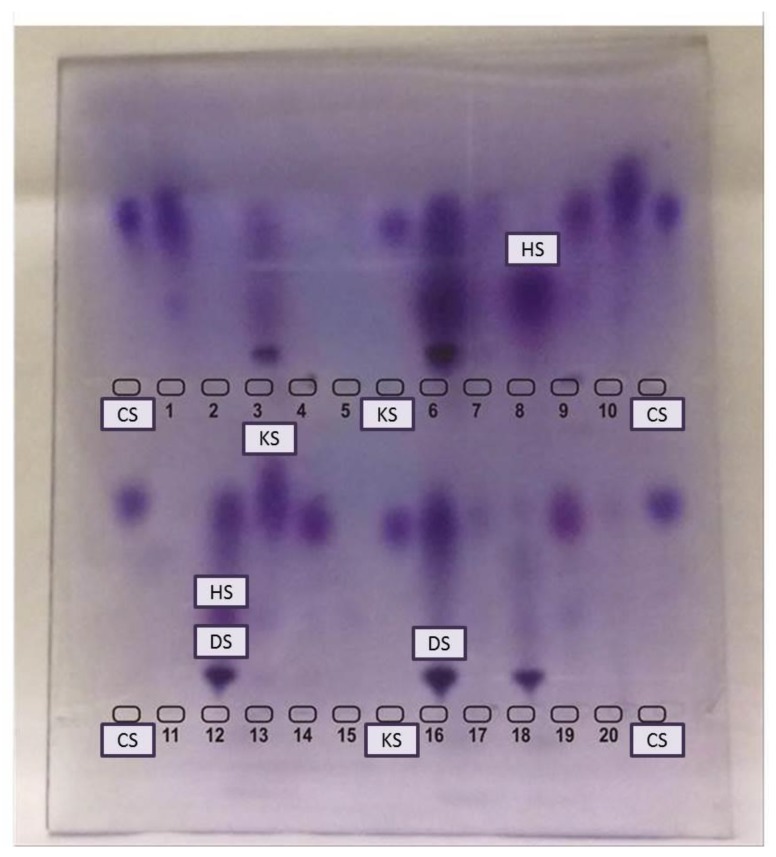
Electrophoresis of urinary glycosaminoglycans. CS: chondroitin sulfate, HS: heparan sulfate, KS: keratan sulfate. Top wells: 1, 2, 4, 5, 7, 8, 10, 11, 13, 15, 17 & 20 are not suggestive of MPS. 3, 16 & 18: Patients with DS suggestive of MPS VI (but confirmation with enzyme assay is needed). 6 & 12: Patients with DS and HS (perform enzyme assay for MPS I, II, and VII). 9, 14 & 19: patients with KS (perform enzyme assay for MPS IVA & IVB).

Urinary GAGs (uGAGs) analyses with dimethyl methylene blue (DMB) (Figure 1) and electrophoresis (Figure 2) have been the most used biomarkers for MPS [35,36,37,38,39]. This marker has also been used with reliable results as a surrogate marker in clinical trials of enzyme replacement therapy (ERT) [40,41,42,43]. Careful evaluation should be performed of the long-term measurements of GAGs due to influence of age (Figure 1), anthropometric variables, renal function, phenotype correlations, and potential causes for false-negative results [32,39,44,45,46]. KS also might be used as a marker for skeletal dysplasia [47].

**Figure 3:**
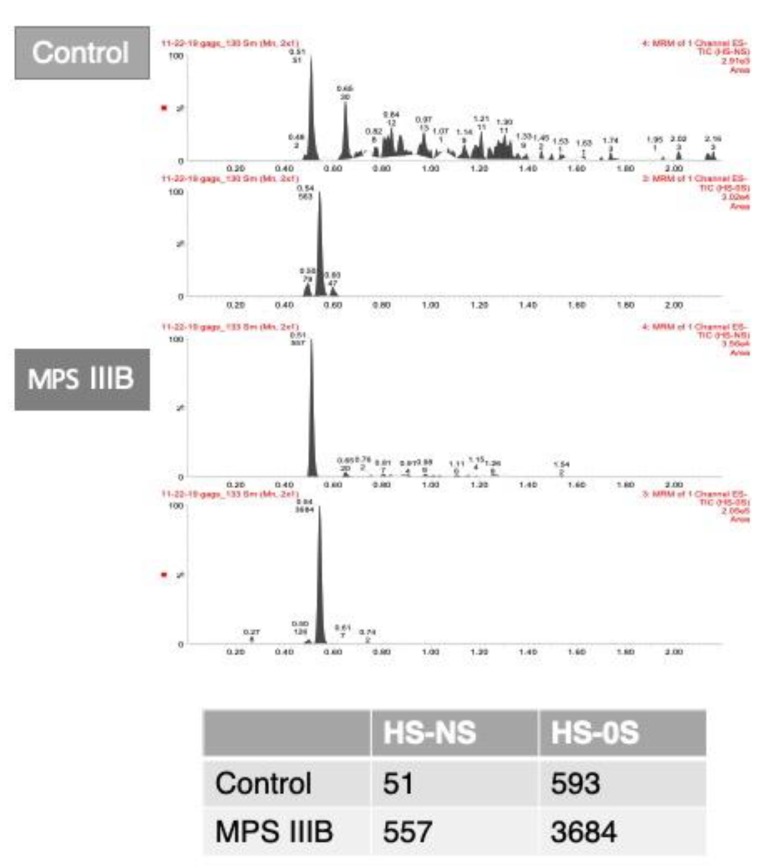
A. Chromatograms of glycosaminoglycans analyzed in dried blood spots from liquid chromatography tandem mass spectrometry in dried blood spots of a control and an MPS IIIB patient. B. Table shows the area counts of the chromatograms. HS-NS: heparan sulfate (0S and NS); MPS IIIB: mucopolysaccharidosis type IIIB.

To quantify GAGs in different matrices, liquid chromatography tandem mass spectrometry has been used [48]. GAGs can be quantified in: urine [34,49,50,51,52,53,54], serum/plasma [34,48,54,55,56], dried blood spots [57,58,59] (Figure 3), amniotic fluid [60], cerebrospinal fluid [34,61], cultured cells [34], and tissues [62]. Some of these assays can be used for newborn screening of MPSs [57,63] or even to allow the discrimination of specific disease subtypes [53]. 

Besides the use of direct markers by analysis of GAGs, indirect markers are also useful. These are molecules that are not the primary storage material, but affect cells, tissues or organs due to the primary storage [32]. Fibroblast growth factor-2 (FGF-2) is a molecule with high affinity for HS [64], thus it can be useful for HS detection [65]. 

The heparin cofactor II-thrombin complex (HCII-T) is also affected by GAG metabolism [32,66]. It has been shown that when DS levels are elevated, HCII-T levels are also elevated [67]. HCII-T is also elevated in the serum of MPS I, II, III, IV and VI patients [68]. This marker has also been used for long-term ERT studies in MPS I, II and MPS VI patients [67,69,70]. The biomarker levels are affected by high-titers of antibodies [67,71].

Another biomarker that can be used is dipeptidyl peptidase (DDP) IV (CD26). Surface-enhanced laser desorption/ionization time of flight (SELDI-TOF) mass spectrometry showed the elevation of DDP-IV in serum of MPS patients, followed by a reduction post-bone-marrow-transplantation (BMT) or ERT [72,73].

The lysosomal impairment seen in the MPS due to the GAG storage also leads to a complex dysfunction that affects secondary markers from cascades downstream, such as glycosphingolipids, phospholipids, and cholesterol [74,75,76,77,78,79]. Glycosphingolipids (GSLs) such as the gangliosides GM1 and GM3 can be used as markers for CNS impairment [75,77,80]. These GSLs have also been used as markers for therapeutic efficacy post-gene-therapy [81] and post-intrathecal-ERT [82]. Bis (monoacylglycerol) phosphate (BMP) is a phospholipid located within the endosomal/lysosomal membrane that contributes to the degradation of glycosphingolipids and transportation of cholesterol. MPS I, II and IIIA patients presented higher plasmatic levels of BMP compared to controls [83]. 

It is also well established in the pathophysiology of MPS that the progressive GAG storage leads to constant inflammation and immune responses, furthermore, several inflammatory and oxidative stress markers can be used as biomarkers for MPS [84,85,86,87,88,89,90,91,92]. 

Nonetheless, several biomarkers have been proposed for the diagnosis and follow-up of MPS patients. However, no biomarker has yet been truly elevated or reduced post-treatment for all MPS subtypes. uGAGs have been widely used, but they do not directly correlate to the clinical impairment and still have limitations for some MPS subtypes. Furthermore, no biomarker can predict and discriminate between severe phenotypes, although great progress has been made with the quantification of GAGs in the CSF with correlations with brain magnetic resonance imaging (MRI). However, long-term studies are still required. 

## 4. Enzyme Assays

In several cases, the biochemical investigation of an MPS started with the analyses of glycosaminoglycans (GAG) in urine before the enzyme assay; this is performed because the GAG storage is due to a primary defect in the enzymatic activity. This analysis in urine can then drive more robust specific enzyme assays, saving time and costs. The GAG monitoring also offers the advantage of treatment monitoring.

For post-natal evaluations, the sample types that are most used are the dried blood spots (DBS), plasma, leukocytes, and fibroblasts, while, for the pre-natal investigations, chorionic villi and amniotic fluid are most used. In the post-natal period, the gold standard is the quantification of enzymes in leukocytes or fibroblasts, and the results for DBS require confirmation [4,93].

Fibroblasts offer the advantage of minimizing the variation effects in temperature and shipping due to the cell culture in the lab. The use of fibroblasts also means a larger amount of sample in which a larger number of cells can be cultured and assays can be repeated, without the need for a new sample collection. The disadvantages of the use of fibroblasts are related to the invasive collection of skin biopsies and a longer turnaround time for the results (cell culture usually takes about 2 to 4 weeks), and sometimes there is a risk of contamination with no cell growth. This procedure is also more expensive. Leukocytes from whole blood are an alternative to the enzyme assay without the need for cell culture, thus it allows a faster turnaround time of less than 2 weeks. This is very useful, for example in a post-natal investigation of families with a history of MPS I. In most cases, leukocyte samples provide enough cell counts for the analyses, or, if cells are not enough, a new sample collection might be requested [94]. Furthermore, leukocyte samples are highly susceptible to temperature variations, which are a big issue in tropical countries. To avoid sample deterioration, it is usually recommended that samples must arrive in the laboratory within 24 to 48h post-draw [95].

Plasma samples may be useful for the assay of some MPS enzymes (associated with MPS I, MPS II, MPS IIIB, and MPS VII) when leucocytes cannot be obtained, especially when the sample cannot be shipped immediately after collection. In this case, plasma could be obtained and stored frozen until shipment is possible. Generally, leukocytes are preferable as a more reliable source of enzymes. However, after careful usage of plasma for MPS I and VII evaluation, we have found false positives.

DBS is a very interesting alternative, mainly for several regions where the shipment of blood or skin biopsies is a challenge; and it is already the sample of choice for newborn screening programs [5]. However, as described for leukocytes, proper sample collection and shipping are critical for the success of the analysis. The date of collection should always be written on the card to aid the interpretation of results. The card should be dried at room temperature for at least 4 h. Due to the temperature-sensitive nature of some enzymes in a DBS [96], cards should be stored at 4°C after drying and shipped as promptly as possible; the longer the period of time between collection and analysis, the higher the risk of a false positive result. There is no doubt about the power of the use of DBS in enzyme assays for screening, nonetheless, this sample is still not considered a gold standard, such as fibroblasts or leukocytes, because there is a smaller number of cells per spot. There is also a need for further studies with regards to the stability of the enzyme activity in DBS, especially for samples exposed to longer shipping times and increased temperatures. Thus, positive results in the DBS screening should be confirmed in leukocytes and or fibroblasts [94]. 

The most common reaction employed is based on the quantification of enzyme activity in biological fluids through catalysis. In several methods for lysosomal enzyme quantification, there is the use of endpoint quantification that is then determined by the substrate or product concentration at a specific timepoint after sample addition. Most enzyme assays for lysosomal disorders diagnosis rely on spectrofluorometry, which uses enzyme-specific substrates with a fluorogenic radical (4-methylumbelliferyl) to generate a fluorophore product that will absorb energy at a specific wavelength and then emit it at another, longer wavelength to determine the quantity of product produced. Spectrophotometry is also a widely used technique based on chromophores (as p-nitrochatechol sulfate specific for arylsulfatase B) that excite themselves and emit colors depending on the energy released by the change from the basal to the excited state [2] (Table 3). 

To assure the quality of the enzymatic assays, it is important to always include positive and negative controls to analyze an additional enzyme, to confirm the integrity of the sample. There is also the possibility of multiple sulfatase deficiency (MSD), in which the reference enzyme should be a sulfatase to perform the differential diagnosis. With regards to mucolipidosis II/III (ML II/III) lysosomal enzymes are not targeted to specific cell types and excreted in the extracellular matrix, leading to high enzyme activity in plasma and DBS and low activity in fibroblasts. Nonetheless, careful examination must be taken in fibroblasts because the finding of an enzymatic defect does not exclude the possibility of MLII/III [93]. 

Specific enzyme deficiencies are associated with each MPS subtype. Some patients have very low enzyme levels in vitro, but normal levels in vivo; this is known as pseudodeficiency due to genetic polymorphisms affecting the activity of one of these lysosomal hydrolases in the in vitro testing, but it is not significant for the in vivo GAG degradation. The potential presence of an enzymatic pseudodeficiency poses a limitation for enzymatic tests and should be investigated whenever the results from an enzymatic assay do not concur with the clinical phenotype of the patient. Pseudodeficiency has been reported for MPS I, IIIB, IVB, VI, and VII, usually associated with specific gene variants [97].

It is important to emphasize that the residual levels of the enzyme are not related to the phenotype or disease severity. Biochemical tests in urine (GAG assays) or in cells (enzyme reactions) elucidate the diagnosis, but molecular testing is very important to characterize the disease and, in some cases, it aids the phenotype prediction [4], which is also important to rule out pseudodeficiency.

## 5. Molecular Genetics Analyses

Although enzyme activity assay is considered the gold standard for the diagnosis of MPS disorders, molecular genetic testing is recommended [98] and, whenever possible, diagnostic conclusions should be made taking the clinical, biochemical, and molecular genetics results into consideration.

Molecular analysis is helpful for: (a) the confirmation of an MPS diagnosis when an enzyme assay in leukocytes or fibroblasts is not possible, as a diagnosis cannot depend only on urinary GAGs and/or enzyme assay in DBS [99]; (b) the confirmation of an MPS diagnosis when the results of enzyme activity analysis are not clear (especially when a high residual activity is observed, or when the sample conditions for enzyme assay are not ideal); (c) in cases with low enzyme activity and normal urinary GAGs, where molecular analysis is required to discriminate pseudodeficiency, carrier status and normal status [100]; (d) Phenotype prediction, which may be important for management decisions as some mutations have been associated with milder phenotypes—MPS I (p.Ser633Trp), MPS IIIA (p.Arg206Pro, p.Ser347Phe/p.Asp444Gly and p.Glu369Lys/p.Pro128Leu), MPS IIIC (p.Gly262Arg and p.Ser539Cys)—while others have been associated with the severe phenotype—MPS I (p.Trp402Ter and p.Gln70Ter), MPS II (p.Ser333Leu and *IDS* total gene deletions), MPS IIIA (p.Arg433Gln) [101,102]—(e) identification of the suitability of the patient to a mutation-specific therapy, such as stop codon read-through [103,104,105]; (f) prenatal diagnosis, alternatively or in addition to biochemical diagnosis [106,107]; (g) diagnosis of MPS-like syndromes (such as MPSPS, which results from non-enzymatic lysosomal protein deficiency, and therefore has increased GAG excretion with normal activity of the MPS-related enzymes.)

There is a high allelic heterogeneity among genes associated with MPS, which is the main cause of the wide spectrum observed in these disorders and cannot always be correlated with residual enzyme activity [108]. To date, more than 2200 mutations have been reported in all 11 genes related to MPS, with the majority of individuals showing private mutations (~70%) [109,110]. This broad mutational spectrum is composed mostly by missense/nonsense variants (64.6%), followed by small deletion/insertions/indels (19.6%), splicing defects (8.1%), complex rearrangements (4%), gross deletion/insertion/indels (3.6%) and defects in regulatory regions (0.2%) (http://www.hgmd.cf.ac.uk/ac) (Table 4).

Different molecular approaches for rapid detection of disease-causing mutations are available, each one with its own indications and limitations [102]. Sanger sequencing remains the gold standard method for the identification of genetic variations (point mutations and small insertions and deletions) in these monogenic disorders. However, due to the high level of allelic heterogeneity and the fact that this methodology can only analyze one DNA segment/exon at a time, it is a labor-intensive, time-consuming and expensive process. Currently, this methodology is used to investigate subjects in a family with a known mutation in a specific MPS gene.

In general, sequence analysis has the potential to detect pathogenic variants in 88.2 to 98.8% of probands with the MPS phenotype, mostly point mutations and small insertions/deletions. For the detection of other types of variants, such as complex rearrangements, there are methods based on gene-targeted deletion/duplication analysis as quantitative polymerase chain reaction (qPCR), long-range PCR, Multiplex Ligation-Dependent Probe Amplification (MLPA), gene-targeted microarray designed to detect single-exon and multi-exon deletions or duplications that can be used to complement the molecular strategy. 

Furthermore, new technologies, such as next-generation sequencing (NGS), are becoming more accessible and relatively affordable for the MPS diagnostic routine. This technology was revealed as a powerful approach to overcome the wide clinical and genetic heterogeneity of MPS, allowing the simultaneous screening of several MPS-related genes with shorter turn-around times for the final report. NGS applications include the sequencing of a PCR-amplified set of specific genomic regions (NGS gene panel or Targeted NGS, TNGS), and the sequencing of whole exome (WES) and whole genome (WGS). 

The TNGS of MPS-associated genes is an interesting option for mutation detection in terms of cost and availability and could be the best option if the clinical and biochemical findings point towards a particular MPS type/subtype, increasing diagnostic yield. One of the advantages of this approach, compared to whole-exome and whole-genome analysis, is that less effort in terms of bioinformatics and computational power is required, since a significantly lesser amount of data is analyzed. The previous results of three custom panels designed to amplify the coding regions of 11 MPS-associated genes demonstrated the high sensitivity and specificity of a TNGS approach to mutation identification when compared to the gold standard (Sanger sequencing), leading to the detection of 250 variants and a 90% breadth of coverage of the targets [7].

In this scenario, WES/WGS could be valuable diagnostic tools: (a) to find novel genes associated with rare conditions, as the newly discovered MPS like syndrome (MPSPS) [111,112]; (b) to expand the recognized phenotypic spectrum of a well-known disease [113,114,115,116]; (c) to elucidate complex phenotypes, as reported by the group of Kaissi et al., when the genetic confirmation of MPS genes involved in a pair of siblings adds to the obscure nature of the disease [117] and (d) as a first-tier diagnostic tool for MPS, with subsequent traditional biochemical testing (GAG quantification and enzyme assay) to confirm molecular diagnosis, in an inversion of the traditional diagnostic algorithm, which may be a trend for the future if the cost of sequencing and the number of laboratories that continue to perform sophisticated enzyme assays continues to decrease [118]. 

Ethical aspects are one of the main challenges of WES/WGS due to incidental findings, such as the identification of pathogenic mutations in genes not related to the main investigation [119]. Careful consideration will also need to be given to variants of unknown significance (VUS), identified through NGS. An in-depth analysis of this type of variant should be taken into consideration to decide the most appropriate clinical management.

## 6. Newborn Screening

Newborn screening is extremely powerful for conditions that are not too rare and whose patients are usually asymptomatic at birth. MPSs are progressive, debilitating, and often life-threatening conditions. The correct diagnosis for these conditions usually takes several years, in what is known as the “MPS odyssey”, and treatment is already approved for several of the MPS subtypes. Thus, NBS for MPS is tremendously important once early diagnosis leads to early intervention, which could make a significant difference in the patient’s outcomes and prevent debilitating manifestations [120]. 

**Figure 4:**
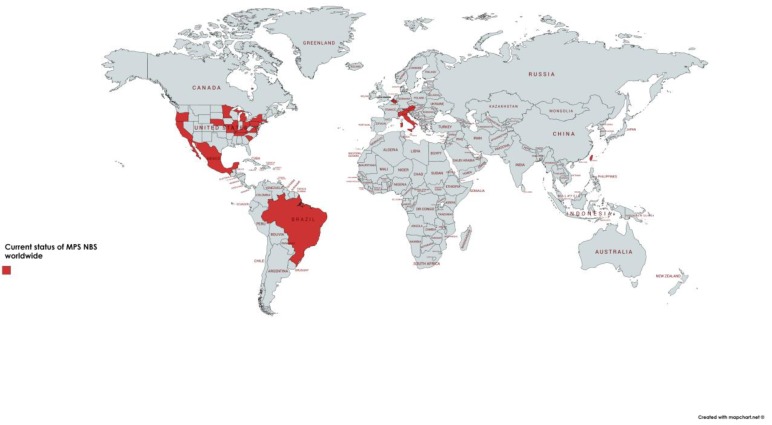
Map with the regions where screening is regular or in pilot stages. Currently, several states of the United States of America (USA) are universally screening for MPS I, and the state of Illinois (IL) is also screening for MPS II. Some centers in Italy are conducting screening for MPS I and Taiwan is screening for MPS I with pilot studies for MPS II and VI. IL: Illinois, MPS I: mucopolysaccharidosis type I, MPS II: mucopolysaccharidosis type II.

Since 2016, MPS I has been officially added to the recommended uniform screening panel (RUSP) in the United States, allowing several states to universally screen for this disorder, while others are still in preparation [121,122,123,124] (https://www.newsteps.org/resources/newborn-screening-status-all-disorders) (Figure 4). The state of Illinois, in the USA, is also universally screening for MPS II, in addition to MPS I [125]. Screening for MPS is also routine in Taiwan, including MPS I, II and VI [126,127] and in some regions of Italy [120,128]. Pilot studies for screening of MPS, mainly for MPS I, were performed in Austria [129], Belgium [130], Brazil [100], Mexico [131] and few other countries.

The current major methodologies employed for the screening of MPS are the quantification of the lysosomal enzymes by digital microfluidics (DMF) with currently available assays for MPS I and II, although this platform is limited by the number of enzymes that can be multiplexed in a single assay [132,133,134]. On the other hand, tandem mass spectrometry has been widely used for the NBS of MPS I and now it is available for the screening of MPS II, IIIB, IVA, VI, and VII [135]. 

Another strategy that has been employed for pilot studies of MPS I, II and III is based on the quantification of GAGs as a first-tier, followed by a second-tier assay for enzyme quantification [57,58]. Despite the fact this is a very useful assay, the false positive rates were much higher than with enzyme assays as the first-tier [122]. Thus, this approach is now employed for the confirmation of cases whose enzyme assays are abnormal [120,136,137]. 

Now that multiplexing assays are available for the screening of MPS I, II, IIIB, IVA, VI, and VII, more centers are expected to start pilot studies for several of the MPSs. Molecular assays can also be performed by next-generation sequencing (NGS) allowing for the identification of variants, but careful interpretation should be performed with variants of unknown significance. With the advent of high-throughput screening methods and the pressure from advocacy groups, it is likely that MPS NBS will soon become a reality. This will also enable reproductive decisions and help genetic counseling. The impact that diagnosing and treating an MPS patient has in the family is tremendous: early intervention can slow down disease progression and improve quality of life for the patient and their family [138].

## 7. Diagnostic Work-Up 

We propose that for every patient with suspected MPS, samples of urine (typically 15–20 mL) and of EDTA blood (typically 8 mL, or two purple cap tubes) are obtained. Urine should be kept frozen until processed. The blood should be kept in the fridge (4–8 °C until shipped to the laboratory, which should occur as soon as possible). When the blood arrives at the diagnostic laboratory, leukocytes should be isolated, plasma should be obtained and DBS should be prepared. The leukocyte pellet and the plasma should be kept frozen, and the DBS should be allowed to dry at least for 4 h and then it should be kept in the freezer (–20 °C) in an individual plastic bag, preferably with desiccant. If the sample will not be able to arrive at the diagnostic lab in 3–4 days, plasma and DBS should be obtained after collection (plasma should be kept in the freezer and DBS in the fridge, until shipment to the diagnostic laboratory). 

**Figure 5:**
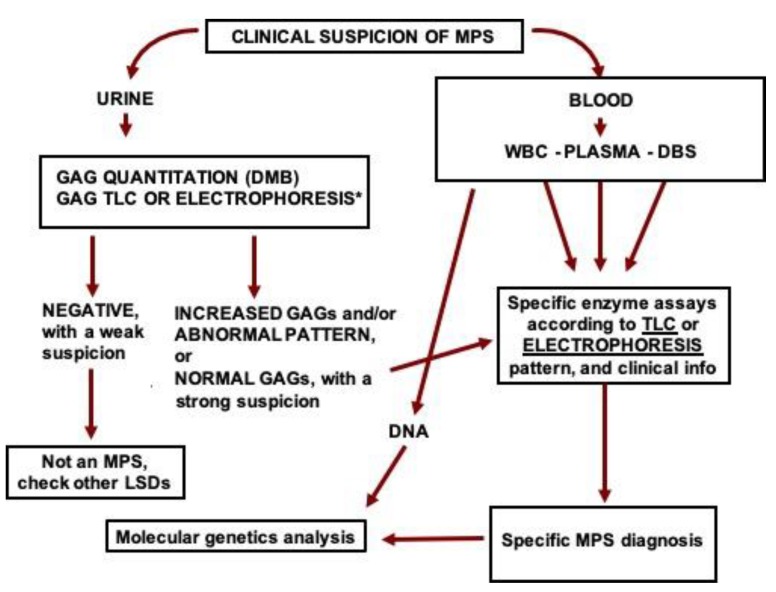
Proposed flow-chart for the investigation of MPS in high-risk patients. GAG: glycosaminoglycans, DMB: dimethyl methylene blue, TLC: thin-layer chromatography, MPS: mucopolysaccharidosis, WBC: white blood cells, DBS: dried blood spots, DNA: deoxyribonucleic acid. *GAGs can also be analyzed by liquid chromatography tandem mass spectrometry.

The diagnostic lab could start the investigation by measuring urinary GAGs and identifying the GAG species present in the urine. If GAGs are increased and/or GAG pattern is abnormal, blood samples should be retrieved from the fridge or freezer and processed for the measurement of the activity of specific MPS enzymes, according to the GAG results and clinical suspicion, which usually leads to the diagnosis of the specific MPS type and enables specific therapy to be introduced whenever available. Thereafter, DNA could be obtained from the blood (leukocytes or DBS) and the specific gene sequenced. With the mutations identified, the family could benefit from phenotype prediction, carrier identification, genetic counseling, and prenatal diagnosis. A summary of the proposed diagnostic flow-chart is presented in Figure 5.

When the enzyme assays in leukocytes (or fibroblasts) is not possible, it is recommended to postpone the diagnosis confirmation in the cases that present urinary GAGs and/or enzyme assays in plasma or DBS until the results of the molecular analysis are available. When urine is not available, it is recommended to measure GAG species in DBS by tandem mass spectrometry, to have a demonstration of the functional impact of the enzyme deficiency and/or mutation profile. 

Babies tested in the newborn screening programs who present decreased enzyme activity should have, due to the high prevalence of pseudodeficiency, GAG species measured and a genetic analysis performed in the blood sample before the suspicion of an MPS diagnosis is taken to the family. 

The increased use of NGS as a primary investigation method, especially when WES and WGS are performed, frequently leads to the finding of genetic variants in the MPS genes, even in patients who were not primarily investigated due to an MPS suspicion. We recommend that, in these cases, enzyme measurement and GAG analyses are performed to allow the estimation of the pathogenicity of the variants identified. This is very important, as NGS has become the first method in the investigation process. 

With specific therapies already available for most MPS patients, and with clinical trials in progress for many conditions, the specific diagnosis of MPS as early as possible is becoming increasingly necessary. 

## Figures and Tables

**Table 1:** Signs and symptoms that should raise clinical suspicion of MPS.

Sign/Symptom of MPS		MPS Types							
		I	II	III	IV	VI	VII	IX	Plus
**Head and neck**	Coarse facial features	+	+	+	+	+	+		+
	Hypertrichosis/thick scalp hair	+	+	+		+	+		+
	Hearing loss	+	+	+	+	+	+		+
	Macrocephaly/scaphocephaly	+	+	+		+	+		
	Corneal clouding	+			+	+	+		
	Abnormal dentition	+	+	+	+	+	+		
	J-shaped sella turcica	+	+			+	+		+
**Osteoarticular**	Short stature	+	+		+	+	+		+
	Joint stiffness	+	+	+		+	+	+	+
	Hip dysplasia	+	+	+	+	+	+	+	
	Thoracolumbar kyphosis	+	+		+	+	+		+
	Genu valgum	+	+		+	+	+		
	Odontoid dysplasia	+	+		+	+			
	Claw hands	+	+			+	+		+
	Bullet-shaped phalanges	+	+			+	+		+
	Carpal tunnel syndrome	+	+			+			
	Joint laxity				+				
**Cardiovascular**	Valve thickening / dysfunction	+	+	+	+	+	+		
	Left ventricular hypertrophy	+	+	+	+	+	+		+
**Neurological**	Developmental delay/intellectual disability	+	+	+			+		+
	Ventriculomegaly	+	+	+		+	+		
	Dilated perivascular spaces	+	+	+		+			
	Hyperactive / aggressive behavior		+	+					
**Airways**	Recurrent respiratory infections	+	+	+	+	+	+		+
	Obstructive airway disease	+	+		+	+	+		+
**Abdomen**	Hepatomegaly/splenomegaly	+	+	+		+	+		
	Umbilical/inguinal hernia	+	+	+			+		
**Others**	Abnormal granulation in leukocytes	+	+	+	+	+	+		+
	Fetal hydrops	+			+		+		
	Proteinuria								+
	Cytopenias								+

A plus sign (+) indicates that the manifestation is associated to that specific mucopolysaccharidosis (MPS) disorder. MPSPS: mucopolysaccharidosis-plus syndrome.

**Table 2:** High-risk groups for MPS.

Phenotype	Main Types of MPS	Differential Diagnoses
**“Hurler-like phenotype” (Coarse facial features, hepatosplenomegaly, dysostosis multiplex and claw hand deformities)**	I, II, VI, VII and MPSPS	Multiple sulfatase deficiency, GM1 gangliosidosis, Galactosialidosis, Mucolipidosis, Oligosacaridosis
**Progressive joint disease with childhood onset**	IX; attenuated forms of other types of MPS	Camptodactyly-arthropathy-coxa vara-pericarditis syndrome, Blau syndrome, Progressive pseudorheumatoid dysplasia, Multicentric carpotarsal osteolysis syndrome, Czech dysplasia
**Nonimmune hydrops fetalis**	I, IV and VII	Malformations, Chromosomal disorders, other LDs, infections, skeletal dysplasias
**Developmental delay/regression and Hyperactivity/aggressive behavior**	III	Several other metabolic, genetic and acquired causes of mental retardation
**Spondyloepiphyseal dysplasia**	IV	Dyggve-Melchior-Clausen dysplasia and other spondylo-epi(meta)physeal dysplasias

GM1: gangliosidosis type I; LDs: lysosomal disorders; MPSPS: mucopolysaccharidosis-plus syndrome.

**Table 3:** Diagnosis of each MPS according to the methodology and sample types.

MPS Type *	Deficient Enzyme	Methods	Samples
MPS I	α-L-iduronidase	Spectrofluorometry, MS/MS, DMF-F	L, F, DBS, CV, A
MPS II	Iduronate-2-sulfatase	Spectrofluorometry, MS/MS, DMF-F	L, F, DBS, CV, A
MPS IIIA	Heparin sulfamidase	Spectrofluorometry, MS/MS	L, F, CV, A, DBS
MPS IIIB	N-acetylglucosaminidase	Spectrofluorometry, MS/MS	L, F, DBS, CV, A
MPS IIIC	N-acetyl-transferase	Spectrofluorometry	L, F, CV, A*
MPS IIID	N-acetylglucosamine-6-sulfatase	Spectrofluorometry	L, F, CV, A*
MPS IVA	N-acetylgalactosamine-6-sulfatase	Spectrofluorometry, MS/MS	L, F, DBS, CV, A
MPS IVB	ß-galactosidase	Spectrofluorometry	L, F, DBS, CV, A
MPS VI	Arylsulfatase B	Spectrophotometry, MS/MS	L, F, DBS, CV, A
MPS VII	ß-glucuronidase	Spectrofluorometry, MS/MS	L, F, DBS, CV, A

L: leukocytes, F: fibroblasts, CV: chorionic villi, A: amniocytes, MS/MS: tandem mass spectrometry, DMF-F: digital microfluidics methods use DBS as sample. No published results for the analysis of MPS IIIC and D in dried blood spots (DBS) by MS/MS until this moment. * MPS IX has a peculiar presentation and is not usually searched in the diagnostic work-ups.

**Table 4:** Review of disease-causing mutations in the Mucopolysaccharidosis.

Disorder	Gene	Chr.	Pathogenic Variants Reported *	Mutation Type (%)					
				M/N	S	R	SD/SI/SID	GD/GI/GID	CR
MPS I	*IDUA*	4p16	292	56.9	15.8	0.3	23.6	2.4	1
MPS II	*IDS*	Xq28	659	49.8	9.3	0	29.1	8.8	3
MPS IIIA	*SGSH*	17q25	150	76.6	2	0	18.7	2.7	0
MPS IIIB	*NAGLU*	17q21	177	67.3	4.5	0	23.7	4.5	0
MPS IIIC	*HGSNAT*	8p11	72	55.6	19.4	0	16.7	6.9	1.4
MPC IIID	*GNS*	12q14	25	28	16	0	40	8	8
MPS IVA	*GALNS*	16q24	348	74.4	9.8	0	11.5	3.4	0.9
MPS IVB	*GLB1*	3p21	234	76	7.3	0	15.4	1.3	0
MPS VI	*ARSB*	5q14	208	76	5.2	0	15.4	3.4	0
MPS VII	*GUSB*	17q21	66	81.8	7.6	1.5	7.6	1.5	0
MPS IX	*HYAL1*	3p21	3	33.4	0	0	33.3	0	33.3
MPSPS #	*VPS33A*	12q24	1	100	0	0	0	0	0
**Total**			2235	64.6	8	0.2	19.6	3.6	4

Chr: chromosome, M/N: missense/ nonsense, S: splicing, R: regulatory, SD: small deletions, SI: small insertions, SID: Small indels, GD: gross deletions, GI: gross insertions, GID: Gross indels, CR: complex rearrangements. # MPSPS: mucopolysaccharidosis-plus syndrome; *: HGMD professional 2019.1 (accessed on: August 15 2019).
